# Expression Profile Analysis and Image Observation of miRNA in Serum of Patients with Obstructive Sleep Apnea-Hypopnea Syndrome

**DOI:** 10.1155/2021/9731502

**Published:** 2021-12-14

**Authors:** Haiyan Shao, Peihong Shen, Junfeng Chen

**Affiliations:** ^1^Department of Respiratory and Critical Care Medicine, The First People's Hospital of Wenling, Taizhou, Zhejiang 317500, China; ^2^Department of Integrated Traditional and Western Medicine, The First People's Hospital of Wenling, Taizhou, Zhejiang 317500, China

## Abstract

The expression profile and image observation of miRNA in serum of patients with obstructive sleep apnea-hypopnea syndrome were investigated. Bioinformatics methods were used to explore the molecular mechanism of obstructive sleep apnea-hypopnea syndrome (OSAHS)-related hypertension and explore the differentially expressed core miRNAs and regulatory factors, providing a theoretical basis for seeking molecular targets for clinical diagnosis and treatment. The miRNA datasets of patients with OSAHS and those with hypertension were downloaded from the public database to obtain differentially expressed miRNAs and explore the biological processes and pathways involved in the target genes. The core miRNAs and competitive endogenous RNA (ceRNA) transcription factors (TFs) were obtained by database mining and Cytoscape network analysis. The results showed that 2,579 differentially expressed miRNAs were obtained from the GSE112093 dataset. Seven upregulated miRNAs (hsa-miR-7107-5p, hsa-miR-7110-5p, hsa-miR-595, hsa-miR-1268b, hsa-miR-3064-5p, hsa-miR-68565p, and hsa-miR-1180-3p) and one downregulated miRNA (hsa-miR-22-3p) were obtained from the GSE112093 dataset. It is proved that hsa-miR-22-3p, hsa-miR-595, hsa-miR-6856-5pKcnq1ot1, neat1, Tsix, ERG, kdm2b, and Runx1 may be involved in the pathogenesis of OSAHS-related hypertension, which provided a theoretical basis for the mechanism research and clinical treatment of OSAHS.

## 1. Introduction

Obstructive sleep apnea (OSA) is a chronic inflammatory airway disease in which the upper airway repeatedly obstructs completely or partially during sleep at night, leading to obvious hypoxemia, hypercapnia, and sleep structure disorder and then causing a series of clinical symptoms such as cardiocerebrovascular disease and metabolic syndrome. The prevalence of OSA is about 3%–7% in adult males and 2%–5% in females [1]. At present, the incidence of cardiovascular and cerebrovascular diseases secondary to OSA, type 2 diabetes mellitus, and metabolic syndrome is on the rise. In particular, the secondary cardiovascular diseases have brought heavy health and economic burden to patients. Related studies have pointed out that the incidence or mortality of cardiovascular diseases secondary to OSA, including hypertension, endothelial dysfunction, heart failure, and arrhythmia, is increasing, and its difficulty in treatment is still a major challenge. This series of damage is closely related to chronic intermittent hypoxia (CIH) caused by OSA. The process of CIH is similar to ischemia/reperfusion injury (I/R). In this process, a large amount of reactive oxygen species (ROS) are produced, which leads to oxidative stress and a series of inflammatory reactions, resulting in vascular endothelial damage, abnormal activity of the sympathetic nervous system at night, and three major metabolic disorders. The accumulated damage will eventually lead to systemic multiple system and organ damage, such as the heart, lungs, brain, pancreas, digestive system, and blood system. MicroRNA (miRNA) plays an important role in inflammation, oxidative stress, hypoxia, and metabolic disorders [2].

miRNA is a kind of noncoding RNA. Accurate regulation of gene expression is very important for the growth, development, and function of organisms. miRNA is a member of microRNA family, which can negatively regulate target genes [3] by mRNA splicing and protein translation inhibition. First, miRNA genes are transcribed into primary miRNA (PRI miRNA) with stem ring structure in the nucleus through RNA polymerase II (RNA Pol II) or RNA polymerase III (RNA Pol III). Subsequently, PRI miRNA was cleaved by drosha-dgcr8 (Pasha), a microprocessor complex in the nucleus, to obtain a pre-miRNA with hairpin structure and a length of about 70 bases [4]. Then, the pre-miRNA was transferred into the cytoplasm through the export-5-dependent transport mechanism, and then, the mature miRNA with 18–25 base length was formed by the second cleavage of another kind of ribonuclease III (Dicer). The functional chain of mature miRNA was loaded into RNA-induced silencing complex (RISC) together with Argonaute (ago2) protein. The 5′ end of miRNA is complementary to the 3′-UTR of miRNA, and there are two possible ways of complementary pairing: complete complementary binding and incomplete complementary binding. Complete complementation means that the target gene loses its biological effect by degrading the target gene. Incomplete complementation is binding to the 3′ untranslated region of the target gene, resulting in the degradation of miRNA or inhibition of its translation process and regulation of gene expression. It can regulate cell proliferation, differentiation, cell death under physiological and pathological conditions, cell metabolism, and immune response [5]. Different types of miRNA have different functions. In recent years, the research on the function and mechanism of each type of miRNA has been deepened, especially in the cardiovascular diseases associated with OSA, which has been further elaborated [6].

MicroRNA (miRNA) is a class of noncoding small RNA molecules, which can inhibit the expression of specific target mRNA regulation genes. A series of OSAHS-related differential miRNAs have been identified, involving a variety of pathophysiological processes. Some studies have also pointed out that the change in miRNA is closely related to the occurrence and development of hypertension [7]. Therefore, it is speculated that miRNA is differentially expressed in OSAHS patients, and related regulatory factors may induce hypertension through epigenetic regulation. Competing endogenous RNA (ceRNA) can regulate gene expression by competitively binding miRNA, including long noncoding RNA (lncRNA), pseudogene, and circular RNA (circRNA). It is of great significance in the regulation of miRNA [8]. The correlation between OSA and lung adenocarcinoma has also been verified in humans; however, the specific mechanism is still unclear and needs further exploration. There are also many conjectures about the internal mechanism of the relationship between OSA and tumorigenesis. For example, chronic intermittent hypoxia leads to the accumulation of excessive reactive oxygen species in the body, which increases the probability of DNA damage during cell replication, thus increasing the incidence of tumors [9]. Chronic intermittent hypoxia induces HIF-1*α* overexpression. Thus, vascular endothelial growth factor increases vascular proliferation and then stimulates tumor growth. Sleep fragmentation can promote tumor growth and malignant invasion by promoting inflammatory TLR4 signal transduction. It is also speculated that it may be related to the specific exosomes produced by OSA. In this study, the patients with OSAHS were compared with those with hypertension, to explore the molecular mechanism of OSAHS-related hypertension by using the latest multifactor regulatory network analysis and multidatabase mining and provide a theoretical basis for seeking molecular targets for clinical diagnosis and treatment [10].

## 2. Data and Methods

### 2.1. Screening of Differentially Expressed miRNAs

OSA can cause metabolic disorders, inflammation, and oxidative stress. Therefore, relevant studies have been carried out on whether miRNA acts on corresponding target genes in these processes to participate in the occurrence and development of diseases. Some scholars have studied the expression of related miRNAs and related target genes in OSA patients and found that there were 104 miRNAs, such as hsa-miR-485-5p and hsa-miR-107, compared with the control group. The expression of hsa-miR-574-5p and hsa-miR-199-3p showed significant differential expression and acted on 119 potential target genes with great potential and then participated in the pathogenesis of OSA, among which hsa-miR-485-5p, hsa-miR-107, hsa-miR-199-3p, and hsa-miR-574-5p can target the regulation of CAD, CPS1, ATP5L, COX6C, ATP5C1, COX5B, ATP5D, ATP5J, and CYP21A2 genes, which play a key role in metabolic pathways. Among them, CAD plays an important role in the regulation of lipid metabolic pathways, oxidative stress, and hypoxia, and CYP21A2 and hsa-miR-107 may be involved in the metabolism of lipid and lipoprotein pathways. Therefore, these miRNAs associated with OSA may play an important role in the damage of the cardiovascular system.

### 2.2. Exclusion of miRNA Associated with Essential Hypertension

The research object is OSAHS-related hypertension, so literature search and database search are used to eliminate the influence of essential hypertension. In PubMed, the keywords and free words of essential hypertension and miRNA were obtained from MESH, and the related literature and published miRNAs were searched by using essential hypertension, primary hypertension, MicroRNA and mirna, respectively. Meanwhile, the MNDR database was searched to obtain miRNA related to hypertension. Compared with differential miRNAs, miRNAs in both groups were excluded [11].

### 2.3. Target Gene Comprehensive Prediction of miRNA-Target Gene Relationship

The acquisition methods are the experimental method and prediction method, respectively. The experimental methods include (1) reporter assay, qRT-PCR with low flux and high accuracy, and (2) CLIP, microarray with high flux and low accuracy, setting low flux experimental data as strong experimental evidence and high flux experimental data as weak experimental evidence, selecting experimental databases miRTarBase and TarBase and prediction databases targetScan and miRDB, and taking the intersection of weak experimental evidence data and prediction data. The final target gene data were obtained by merging the obtained intersection genes with the strong experimental evidence data [12].

### 2.4. Mining and Network Analysis of Regulatory Factors Related to Differential miRNA

**Figure 1:**
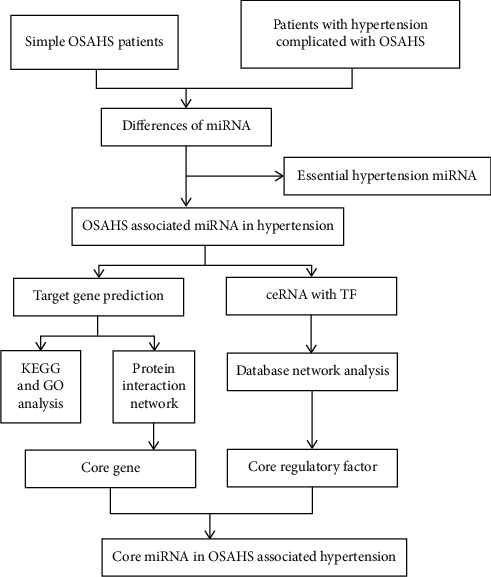
Flowchart of research and design.

1. Data acquisition of competitive endogenous RNA and TF of different miRNAs: lncRNA, circRNA, and pseudogene data related to miRNA were searched in TarBase, and at the same time, experimental data and predicted data were integrated in lncBase database. The screening conditions for the predicted data include the following: the score is adjusted to 0.95, the tissue is set as blood, the specimen is from the nontumor normal group, and the transcription factor (TF) is obtained by using the TransmiR database.
2. ceRNA and TF regulatory network analysis and core factor acquisition: the interaction network of ceRNA and TF with miRNA as the core is constructed by Cytoscape software, and the regulation of each miRNA and the connectivity of nodes are analyzed to determine the core miRNAs and key regulatory factors. At the same time, miRNAs corresponding to the core target genes and key regulatory factors were analyzed to further prove the core miRNAs related to OSAHS. The research process is shown in Figure 1 [13].

### 2.5. miRNA Expression in OSA Patients with Endothelial Dysfunction

Endothelial dysfunction (ED) is an early risk factor for cardiovascular disease, and miRNA-630 plays a very important role in the regulation of endothelial dysfunction in OSA patients. It was found that abnormal expression of 5 miRNAs was found in OSA patients with endothelial dysfunction, among which the expression of miRNA-16-5, miRNA-451A, miRNA-5100, and miRNA-630 was decreased, and the expression of miRNA-4665-3p was increased. The decreased expression of miRNA-630 is particularly important in the process of impaired endothelial function. In the secondary evaluation, the researchers detected that miRNA-630 returned to normal in the exosome of endothelial cells with the recovery of endothelial function after the clinically recommended standard treatment of tonsillectomy and adenoid resection in children with OSA complicated with endothelial dysfunction. In contrast, ED functional phenotypic changes were induced when exosomes were transfected with miRNA-630 inhibitors in subjects without ED.

## 3. Results

### 3.1. Screening Results of Differential miRNA and Query Results of miRNA Related to Essential Hypertension

**Figure 2:**
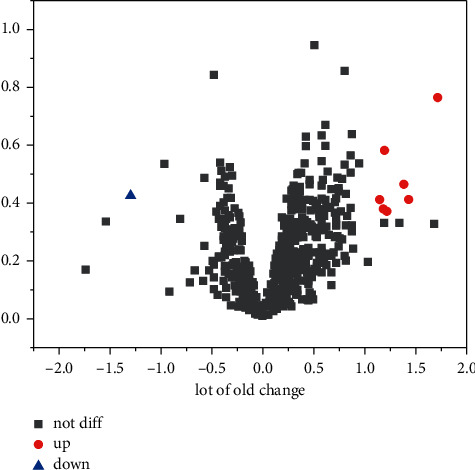
Differential miRNA analysis.

2,579 differentially expressed miRNAs were obtained from the GSE112093 dataset, and 7 upregulated miRNAs were screened by *P* value and differential expression of multiple mRNAs (hsa-miR-7107-5p, hsa-miR-7110-5p, hsa-miR-595, hsa-miR-1268b, and hsa-miR-3066). Analysis of differential miRNA volcano map and comparison of differential miRNA expression are as shown in Figure 2. The miRNA related to essential hypertension obtained from literature search and database query is shown in Table 1. No miRNA was found to duplicate this study [14].

### 3.2. Target Gene Acquisition and GO and KEGG Analysis

**Figure 3:**
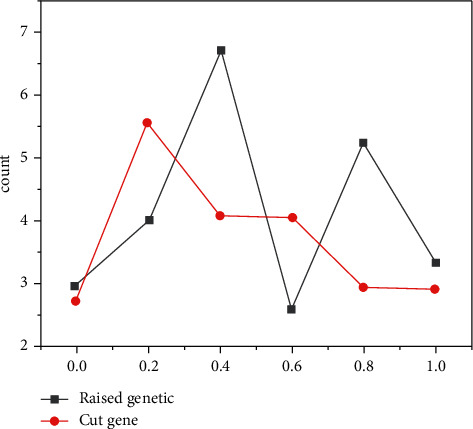
KEGG analysis of target gene.

After comprehensive analysis, it was found that 91 miRNA-related target genes were upregulated and 67 miRNA-related target genes were downregulated. After GO analysis and merger, the top 15 results were listed before sorting by *P* value, and their gene counts were displayed. It is found that biological processes focus on the positive regulation of RNA polymerase II promoter transcription, the positive regulation of sequence-specific DNA binding transcription factor activity, peptidyl-lysine deacetylation, gene expression regulation, monocyte differentiation, cell migration, and so on. Six pathways were obtained after the intersection of two groups of KEGG pathways, and their gene counts and *P* values were displayed (as shown in Figure 3). The visual analysis showed that thyroid hormone signaling pathway, glucagon signaling pathway, PI3K-Akt signaling pathway, gene count, and *P* value were obviously superior to other pathways, which may be the core pathways of the disease [15].

### 3.3. ceRNA, TF, and miRNA Network Analysis

The inflammatory response and oxidative stress induced by OSA can lead to vascular fibrosis, apoptosis, and sympathetic nerve activation and then lead to endothelial dysfunction and vascular remodeling, which play a very important role in the formation of hypertension. And some studies have confirmed that miRNA plays a key role in maintaining metabolic homeostasis and stabilizing blood pressure. Therefore, the study on whether miRNA acts on target genes related to regulating hypertension in OSA complicated with hypertension can help us better understand the mechanism of the disease and provide new targets for their diagnosis and prognosis evaluation.

After comparing ceRNA (lncRNA, circRNA, and pseudogene), TF, and miRNA regulatory network, the key nodes with higher connectivity analysis have the highest and most significant classification. According to the analysis of core genes and regulatory factors, the core miRNAs are hsa-miR-595, hsa-miR-22-3p, hsa-miR-6856-5p, and hsa-miR-3064-5p. lncRNAs are KCNQ1OT1, NEAT1, and TSIX, and core transcription factors are ERG, KDM2B, and RUNX1 [16].

### 3.4. Exosomes and Their Contents miRNA-33b-3p Can Be Ingested by A549 Cells

Exosomes can flow all over the body with blood after cell secretion. In order to verify that exosomes can be taken up by A549 cells, PKH26 and exosomes were incubated together in the study, and this special exosome was incubated with A549 cells. Under the confocal microscope, A549 cells contained special red fluorescence. The results showed that A549 cells absorbed more exosomes. miRNA is a kind of small molecule that can be easily degraded by the environment and contained in exosomes, which can have a certain protective effect on them. To further verify whether miR-33b in exosomes can enter target cells with exosomes, we transferred fluorescence-labeled miR-33b into A549 cells, collected the exosomes produced, and then incubated them with ordinary A549 cells. The incubated A549 cells were observed under the confocal microscope. Red fluorescence was observed, indicating that miRNA entered A549 cells.

## 4. Discussion

In recent years, the development of epigenetics has provided new theories and clues for the study of the etiology and pathogenesis of hypertension. OSAHS-related hypertension has a high incidence and complicated condition, which affects a wide range of people and has important research significance. But the related molecular mechanism is still unclear. In this study, we explored the differential miRNAs of OSAHS-related hypertension. By analyzing the target genes and signaling pathways and constructing the molecular regulatory network, the core miRNAs and regulatory factors that may participate in the pathogenesis of OSAHS-related hypertension were obtained, which provided a theoretical basis for the study of the mechanism of the disease [17].

Cell biological process and signaling pathway play an important role in the occurrence and development of hypertension. In the analysis of target gene GO, it is found that single-cell differentiation and leukocyte migration are involved. At the same time, some studies show that the proportion of immune cells in patients with OSAHS is abnormal, which is related to autoimmune diseases. This study also confirms the relationship between immunity and OSAHS. KEGG analysis indicated that hypoxia-inducible factor (HIF-1) signaling pathway was activated, and HIF-1 was one of the important factors related to hypoxia. The activation of HIF-1 by OSAHS resulted in an imbalance of gene expression of pro-oxidant and antioxidant enzymes, increased the production of reactive oxygen species (ROS), and further promoted the transcription of some genes which were unfavorable to the cardiovascular system, such as endothelin-1 gene, induced arterial inflammation, and vascular remodeling, which led to hypertension. In addition, the analysis also found that differentially expressed miRNAs are also involved in glucagon signaling pathway and thyroid hormone signaling pathway, which are closely related to the occurrence and development of hypertension and often involve cardiac output, vascular regulation, and other physiological processes, which may lead to changes in blood pressure under the disease background of OSAHS [18].

miRNA is at the center of the regulatory network, and these differentially expressed miRNAs may play an important role. hsa-miR-22 is a miRNA highly expressed in the heart, which regulates the expression and function of genes involved in hypertrophy, sarcomere recombination, and metabolic program changes, and plays a certain regulatory role in oxidative stress, cardiomyocyte apoptosis, autophagy, hypertrophy, fibrosis, and regeneration. In ischemic heart disease and heart failure, the physiological processes such as autophagy, apoptosis, and oxidative stress are affected by a single regulation mechanism. The effect of hsa-miR-22 on myocardium may lead to hypertension by regulating cardiac pump function. In addition, miR-22-3p inhibited the proliferation and migration of vascular smooth muscle cells by targeting MECP2, EVI1, and HMGB1 genes. At the same time, it can also regulate arterial intimal hyperplasia. Abnormal vascular smooth muscle function and intimal hyperplasia may induce atherosclerosis, which will affect vascular compliance, leading to hypertension. It is found that miR-595 is involved in lordosis, and mandibular dysplasia is one of the causes of OSAHS. miR-595 may be associated with OSAHS by influencing the airway anatomy. miR-595 is mostly related to cancer-related diseases. Some studies have found that serum miR-595 in patients with heart failure has differential expression. And it was positively correlated with left ventricular hypertrophy index. In addition, miR-595 was differentially expressed in coronary arteries of patients with heart failure caused by different factors. These studies have proved the regulation of miRNA in the cardiovascular system, and OSAHS may affect the expression of miRNA and then change the physiological state of myocardium and blood vessels and induce the occurrence and development of hypertension [19].

Repeated CIH during OSA sleep at night causes oxidative stress, inflammation, and lipid metabolism disorders. miRNA plays an important role in the occurrence of atherosclerosis (AS) and also plays a nonnegligible regulatory role in these processes. Currently, relevant studies have been conducted on the relationship between miRNA and OSA complicated with AS. ceRNA regulates the expression of target gene transcripts by competitively binding miRNA and realizes the common regulation of the pathological process. The widest regulation range in ceRNA is lncRNA. It has been pointed out that KCNQ10T1 is related to acute myocardial injury, arrhythmia, and coronary heart disease. KCNQ10T1 is significantly upregulated in myocardial tissue under the background of diabetes, and the effects of cell scorch and fibrosis are improved after knockout. KCNQ1OT1 may affect the output capacity of the heart through microscopic regulation of myocardium and then affect the blood pressure of patients. NEATI promoted the formation of neointima. After knockout, the proliferation of vascular smooth muscle cells decreased, and neointima formation decreased significantly. In the background of OSAHS, NEAT1 may affect the physiological state of blood vessels and cause fluctuations in blood pressure. ceRNA may play an important role in the development of diseases. Differential miRNA induced by OSAHS may transmit molecular information by regulating ceRNA, change cardiac output and vascular proliferation and state, and then induce hypertension [20].

## 5. Conclusion

This paper mainly explored that exosomes produced during the characteristic IH and SF processes of OSA play an important role in tumor development, cardiovascular disease complications, and insulin resistance, and these effects are closely related to miRNA in exosomes. This provides a new direction for the treatment of OSA patients with multiple diseases. miRNA can regulate different signaling pathways by acting on corresponding potential target genes, thus causing complications related to cardiovascular diseases such as endothelial dysfunction, hypertension, atherosclerosis, and heart failure. Related studies on the diagnosis, treatment, and prognosis assessment of OSA complications in many human diseases, including cardiovascular diseases, are still a major challenge, and more experiments are needed to verify these predictions. With the deepening of research, the clinical application of miRNA will benefit more patients.

## Figures and Tables

**Table 1:** miRNA related to essential hypertension.

Biomarkers	Expression situation	Organization source	PMID
miR-133b	Lower	Blood plasma	21924071
miR-7-5p	Higher	Serum	29069223
miR-26b-5p	Higher	Serum	29069223
miR-26b	Lower	Peripheral blood mononuclear cells	26358152
miR-133a	Lower	Peripheral blood mononuclear cells	26358152
miR-155	Higher	Vein endothelial cells	21234519
miR-21	Higher	Blood	24284386
miR-21	Higher	Peripheral blood mononuclear cells	26358152
miR-499	Higher	Peripheral blood mononuclear cells	26358152
miR-143	Higher	Peripheral blood leukocytes	29609545
miR-510	Higher	Blood	27881848
miR-145	Lower	Blood	24284386
miR-133	Lower	Blood	24284386
miR-1	Higher	Peripheral blood mononuclear cells	26358152
miR-1	Higher	Blood	24284386
miR-296-5p	Lower	Blood plasma	21924071
miR-296-5p	Lower	Blood plasma	21690488
hem-i-112	Higher	Blood plasma	21690488
hem-1-i-1112	Higher	Blood plasma	21924071
l*e*–7*e*	Higher	Blood plasma	21924071
l*e*–7*e*	Higher	Blood plasma	21690488
miR-208b	Higher	Peripheral blood mononuclear cells	26358152

## Data Availability

The data used to support the findings of this study are available from the corresponding author upon request.

## References

[1] Hou J., Zhao L., Yan J. (2019). Microrna expression profile is altered in the upper airway skeletal muscle tissue of patients with obstructive sleep apnea-hypopnea syndrome. *Journal of International Medical Research*.

[2] Arriola-Villalobos P., Benito-Pascual B., Peraza-Nieves J. (2020). Corneal topographic, anatomic, and biomechanical properties in severe obstructive sleep apnea-hypopnea syndrome. *Cornea*.

[3] Zhang T., Pan Y., Lian J., Pang F., Wen J., Luo Y. (2021). Regional characterization of functional connectivity in patients with sleep apnea hypopnea syndrome during sleep. *Physiological Measurement*.

[4] Dutkowska A., Antczak A., Domańska-Senderowska D., Brzeziańska-Lasota E. (2019). Expression of selected miRNA, RAR*β* and FHIT genes in BALf of squamous cell lung cancer (squamous-cell carcinoma, SCC) patients: a pilot study. *Molecular Biology Reports*.

[5] Wang Y., Wang Q., Li Y. (2020). Integrated analysis of mrna-mirna expression in tilapia infected with tilapia lake virus (TiLV) and identifies primarily immuneresponse genes. *Fish & Shellfish Immunology*.

[6] Zhang B., Li B., Qin F., Bai F., Sun C., Liu Q. (2019). Expression of serum microrna-155 and its clinical importance in patients with heart failure after myocardial infarction. *Journal of International Medical Research*.

[7] Sun C., Xu Y., Luo C., Li Q. (2020). Relationship between enuresis and obstructive sleep apnea-hypopnea syndrome in children. *Journal of International Medical Research*.

[8] Sun H., Zhang Y., Wang J., Kong J. (2019). Annals express: correlation of serum meteorin-like concentration with the presence and severity of obstructive sleep apnea syndrome. *Annals of Clinical Biochemistry*.

[9] Shumnalieva R., Kachakova D., Velikova T., Kaneva R., Monov S. (2020). Ab0020 correlation between serum and synovial concentration of il-17a and mirna expression in rheumatoid arthritis patients. *Annals of the Rheumatic Diseases*.

[10] Zhang L., Zhang H., Huang Y., Xi X., Sun Y. (2020). Expression of immune cell markers and tumor markers in patients with cervical cancer. *International Journal of Gynecological Cancer: Official Journal of the International Gynecological Cancer Society*.

[11] Xiaojun Z., Chan W., Hao W., Fang F., Wei X. (2020). 0727 study on the effect of obstructive sleep apnea-hypopnea syndrome onperioperative management inendoscopic sinussurgerypatients. *Sleep*.

[12] Chalkiadaki E., Andreanos K., Karmiris E., Florou C., Papaconstantinou D. (2021). Ganglion cell layer thickening in patients suffering from obstructive sleep apnea–hypopnea syndrome with long mean apnea–hypopnea duration during sleep. *International Ophthalmology*.

[13] Zhao M., Juanjuan L., Weijia F. (2019). Expression levels of microrna-125b in serum exosomes of patients with asthma of different severity and its diagnostic significance. *Current Drug Metabolism*.

[14] Rusek M., Michalska-Jakubus M., Kowal M., Bełtowski J., Krasowska D. (2019). A novel mirna-4484 is up-regulated on microarray and associated with increased mmp-21 expression in serum of systemic sclerosis patients. *Scientific Reports*.

[15] Poka-Mayap V., Balkissou Adamou D., Massongo M. (2020). Obstructive sleep apnea and hypopnea syndrome in patients admitted in a tertiary hospital in cameroon: prevalence and associated factors. *PLoS One*.

[16] Huang Y., Fu X., Luo H., Wang Q., Xu Q. (2020). Expression of serum anti-ganglioside antibody in patients with guillain-barré syndrome and its relationship with clinical features. *Acta Medica Mediterranea*.

[17] Wang X., Liu Y., Tang G., Wang H., Zhao Y. (2019). Effects of low-temperature plasma treatment on pulmonary function in children with obstructive sleep apnea-hypopnea syndrome. *Irish Journal of Medical Science*.

[18] Li N., Liu X., Han L. (2019). Expression of mirna-146b-5p in patients with thyroid cancer in combination with hashimoto’s disease and its clinical significance. *Oncology letters*.

[19] Fontalba-Romero M. I., Lopez-Enriquez S., Lago-Sampedro A. (2021). Association between the mediterranean diet and metabolic syndrome with serum levels of mirna in morbid obesity. *Nutrients*.

[20] Wang S., Wang Z., Wang Q., Cui Y., Luo S. (2019). Clinical significance of the expression of mirna-21, mirna-31 and mirna-let7 in patients with lung cancer. *Saudi Journal of Biological Sciences*.

